# Shining Light into Adolescent HIV Neuroplasticity: A Study of the Prefrontal Cortex Using Functional Near Infrared Spectrometry

**DOI:** 10.12688/f1000research.169799.2

**Published:** 2026-04-24

**Authors:** Sizwe Zondo, Kate Cockcroft, Candida da Silva Ferreira Barreto, Aline Ferreira Correia

**Affiliations:** 1 Psychology, Rhodes University Psychology Clinic, Grahamstown, Eastern Cape, South Africa; 2 Psychology, University of the Witwatersrand Johannesburg School of Human and Community Development, Johannesburg, Gauteng, South Africa; 3 Neuroergonomics & Neuroimaging Lab, Drexel University School of Biomedical Engineering Science and Health Systems, Philadelphia, Pennsylvania, USA

**Keywords:** adolescent HIV, attention, cART, fNIRS, prefrontal cortex

## Abstract

**Background:**

The human immunodeficiency virus (HIV) crosses the blood-brain barrier, compromising cortical networks and cognition. Neuropharmacological intervention in the form of combination antiretroviral therapy (cART) does not reverse cognitive decline in pediatric and adolescent HIV. In search of options to enhance cognition in affected adolescents, we evaluated the effectiveness of attention training.

**Methods:**

The training was evaluated by pairing participants’ outcomes on the Stroop Colour Word Test (SCWT), a test of attention and inhibitory control, to brain hemodynamic responses measured by functional near-infrared spectrometry (fNIRS) both prior to, and after, the training. Changes in oxygenated haemoglobin in the prefrontal cortex during the SCWT were compared in 15 HIV+ participants who received attention training with that of 13 HIV+ participants who did not receive the intervention.

**Results:**

Intragroup analyses showed differences in oxygenated haemoglobin levels between congruent and incongruent conditions of the SCWT only for participants who received the attention training. Specifically, improved behavioural performance on SCWT for the attention group was associated with a significant decrease in oxygenated haemoglobin in the dorsolateral prefrontal cortex, and frontopolar brain areas in the training group. Decreased hemodynamic activation in the training group suggests greater neural efficiency in completing cognitive tasks.

**Conclusions:**

Findings suggest that customised attention training maybe an effective complementary adjunct to cART for children and adolescents living with HIV.

## Introduction

The human immunodeficiency virus (HIV) is a significant global pandemic, with approximately 41 million people living with the virus. South Africa has the highest burden of HIV, with approximately 14% of the population affected by the virus ( HIV/AIDS, 2024). Children and adolescents living with HIV are particularly vulnerable to neurocognitive deficits which result from neuroinvasion by the virus, together with the toxicity of combination antiretroviral therapies (cARTs) ( Yuan & Kaul, 2019). Notwithstanding the improved nature of contemporary cARTs, to reduce cerebral neurotoxicity, neurocognitive deficits typically manifest as difficulties in higher order executive functions, such as sustained attention and working memory ( Milligan & Cockcroft, 2017; Dahmani et al., 2024).

These findings have a neuroanatomical basis since HIV preferentially affects neuronal networks associated with the fronto-striatal cortex, a key node of the central executive network (CEN), implicated with attention and executive functions ( Yu et al., 2019). For example, Chang et al. (2001) investigated blood oxygen level dependency (BOLD) in HIV+ (n = 11; mean age = 41 years; SD = 4.8 years) and HIV unaffected controls (n = 11; mean age = 38 years; SD = 4.8 years) using functional magnetic resonance imaging (fMRI) during the execution of simple and complex attention tasks. In the simple attention reaction task, participants pressed a button in response to a number that appeared at random intervals on a screen, while the complex attention task required participants to respond only when the displayed number was twice as high as the preceding number. The complex attention task placed a greater cognitive load ^1^ on participants, tapping into inhibitory control and working memory. For the simple attention task, the HIV group showed significantly greater BOLD activation in the inferior lateral prefrontal cortex, and parietal lobes compared to controls (p < 0.05). The increased neural activation in both the frontal and parietal regions for the HIV+ group during a simple attention task is proposed to reflect the brain’s compensatory response to neuroHIV. ^2^ Increased BOLD activation within the prefrontal cortex is indicative of greater requirement for ‘top-down’ neuronal resources necessary to manipulate and maintain working memory during cognitively demanding tasks ( Chang et al., 2001). Significantly, as noted by the authors, neuronal hyperactivation was observed alongside preserved task accuracy, but slower processing speed, suggesting compensatory recruitment of additional neural resources to maintain performance beside underlying cognitive deficiency.

Building on their earlier findings, Chang et al. (2004) examined BOLD responses in the visual attention network among HIV+ (n = 18; mean age = 38.2 years; SD = 1.7 years) and HIV-unaffected participants (n = 18; M = 38 years; SD = 2.1 years). Consistent with Chang et al. (2001), HIV+ individuals showed significantly greater activation in the right prefrontal and parietal cortices under increasing cognitive load. This heightened neural activation was interpreted as compensatory recruitment linked to neuronal inefficiency associated with neuroHIV. These converging findings, alongside the limited cognitive benefits of cART, underscore the importance of developing non-pharmaceutical interventions to address HIV-related cognitive impairment.

Cognitive rehabilitation therapy (CRT), grounded in the principle of cortical neuroplasticity, holds potential to reverse cognitive decline in individuals with neuroHIV by promoting adaptive changes in the cerebral cortex ( Crosson et al., 2017). Evidence supports CRT’s effectiveness in improving attention, working memory, and executive function among adolescents living with HIV ( Ikekwere et al., 2021; Fraser & Cockcroft, 2020; Boivin et al., 2019). However, no studies to date have combined behavioural outcomes with objective neural measures, such as fNIRS, to assess CRT efficacy in this population.

The sole study (at the time of writing) to investigate brain plasticity in neuroHIV by pairing behavioural outcomes with objective brain measures is that of Chang et al. (2017). They paired working memory training (via CogMed) with fMRI in adult participants. At one-month follow-up, participants (HIV+: n = 34, mean age = 50.3 years; SD = 1.9. years; HIV-: n = 42, mean age = 52.6 years; SD = 1.7 years), who underwent 25 sessions of working memory training showed statistically significant decrease in BOLD activation when completing working memory tasks (i.e., the n-back task) compared to their performance prior to the training. The HIV group showed statistically significantly decreased BOLD activation in the right middle prefrontal gyrus when completing the 2-back working memory task, which also correlated with improved scores on untrained tasks, namely the Digit-Span Backward and the Spatial Span Forward, suggesting near transfer of skills to other measures of working memory. Six months later, both groups showed BOLD deactivation in the right medial prefrontal gyrus and primary motor area during the 1-back task. In addition, BOLD deactivation was correlated with significant improvement on the Digit-Span Forward working memory task, compared to pretest. The decreased BOLD-fMRI activation is proposed to indicate cortical reorganization associated with improved neural efficiency when completing working tasks of increased cognitive load ( Chang et al., 2017).

Empirical research on the efficacy of CRT in adolescents living with HIV (ALWH), particularly studies combining behavioural and brain-based measures, remains limited ( Benki-Nugent & Boivin, 2019). Such interventions could be especially valuable in low- and middle-income contexts such as South Africa, where they may help alleviate the HIV associated mental health burden. This study thus (1) examined the impact of sustained attention training on behavioural performance (via the Stroop Colour Word Test) and (2) cortical hemodynamic responses (via fNIRS) in HIV+ adolescents receiving attention brain training. The study focused on attention as the primary domain for the rehabilitation as attention represents the foundational process in cognition and supports higher-order cognitive functions such as executive functions ( Yoo et al., 2024). Moreover, attention is consistently reported as a vulnerable in HIV even in the context of preserved global cognition ( Dahmani et al., 2024). Lastly, attention influences neuronal information processing, such that it alters the firing rate of neuronal cells sensitive to distinct cognitive attributes, such as working memory and processing speed ( Chang et al., 2004). It suffices that subtle attentional inefficiencies may serve as behavioural markers of underlying neural alterations sequent NeuroHIV. Specific to the study, the prefrontal cortex was defined a priori, as the primary region of interest due to its central role in attentional processing and cognitive flexibility ( Hogeveen et al., 2022; Menon & D’Esposito, 2022). While prior research suggests brain training may enhance cognitive efficiency and reduce neural activation in relevant regions, no directional hypotheses were proposed for the study, due to the limited existing evidence from a single study with adult participants.

### Research questions

Compared to controls receiving no attention training, do HIV+ participants receiving sustained attention training show significantly improved scores on the SCWT?

Do improvements on cognitive measures on the SCWT correlate with decreased hemodynamic responses in the prefrontal cortex, post training?

## Methods

This project is part of a larger longitudinal pre-and-post-quasi experimental study to examine the effect of brain training within a cohort of children and adolescents living with HIV in South Africa ( Zondo et al., 2024b). Details of the experimental protocol can be found in Zondo et al. (2024a). The current investigation evaluated whether attention training is associated with improved neural efficiency and increased functional connectivity in the CEN, as measured by the SCWT and its correlation with hemodynamic responses in the prefrontal cortex as measured by fNIRS (via changes in oxygenated (HbO) and deoxygenated hemoglobin (Hb) concentration). A repeated measures design enabled us to collect behavioural and neuroimaging data pre- and post-training.

### Participants

Purposive sampling was used to recruit 26 participants from three shelters caring for children living with HIV. All participants (age *M* = 15.28 years, *SD * = 1.94) were diagnosed with HIV ^3^ and were on a course of cART. Exclusion criteria included (a) traumatic brain injury (b) central nervous system-related ailments (e.g., cerebral palsy, meningitis), or (c) learning difficulties. Written informed consent was obtained from the Directors of the shelters and, where possible, from guardians of the children. Assent was obtained from all participants. Ethical approval for this study was granted by Ethics Committee of the University of the Witwatersrand, South Africa [M211073].

Participants were randomly assigned to either the experimental (n = 13) or control group (n = 13) using Research Randomizer Software ( Urbaniak & Plous, 2013). The control group followed a treatment-as-usual approach. One participant in the control group (age = 12 years), was excluded from the behavioural analysis because they did not respond to more than 38% of the trials and repeatedly pressed the same response key for both congruent and incongruent trials on the SCWT post-assessment. The participants’ behavioural pattern suggested lack of engagement and understanding of the assessment task. Table 1 summarises the sample characteristics. Chi-squared tests revealed no significant differences between the groups, by schooling (χ ^2^ = 0.195, *p* = 0.658), there were, however, differences by sex (χ ^2^ = 3.86, *p* = 0.05) and age (χ ^2^ = 3.94, *p* = 0.05). Although all participants were on cART, significant differences were noted in terms of additional medication between the groups (χ ^2^ = 6.01, *p* = 0.01) with three participants in the experimental group on a course of cART and psychotropic medication and one on ADHD medication.

**Table 1.** Demographics characteristics of participants and their comparisons.

Sample characteristics	Treatment group	Control group	χ ^2^	p value
	n = 13	n = 13		
Sex (F/M) ^a^	5/8	9/4	3.86	0.05
Age Range ^b^ (0-13 years/14-18 years)	3/10	8/5	3.93	0.05
Medication (Mood ^c^/ADHD)	2/1	0/0	6.19	0.01
School (Primary/Secondary)	3/10	4/9	0.195	0.658

*Note.*

^a^ Female and Male categories for sex.

^b^ Age categories were based on WHO age range suggestions for children and adolescents.

^c^ Participants were receiving either Risperidol or Citalopram at low dosages.

### Measures

Participants completed a battery of neurocognitive tests pre- and post-training as detailed in Zondo et al. (2024a). Assessments included a demographic questionnaire, the Developmental Neuropsychological Assessment Second Edition (NEPSY-II), the SCWT and the Behaviour Rating Inventory for Executive Function (BRIEF). The psychometric properties, including the validity and reliability of these measures have been confirmed for South African and African populations ( Kusi-Mensah et al., 2022; Rochat et al., 2017; Shuttleworth-Edwards & Truter, 2023).

Functional Near-Infrared Spectrometry (fNIRS) data was acquired using the NIRxSport2 (NIRx, Medical Technologies), a portable continuous wave fNIRS device, which was administered while participants completed the SCWT (described in Zondo et al. (2024a). Specifically, the SWCT took the form of an fNIRS block design ^4^ adapted from Schroeter et al. (2002). Before completing the computerised version of the SCWT, participants completed the pencil and paper version of the test and received feedback to improve test-wiseness. Lastly, this study followed recommendations for best practices in fNIRS research ( Yücel et al., 2021) and the protocol used for data acquisition, optode placement and study montage is described in Zondo et al. (2024a). Briefly, the protocol was inclusive of 10 blocks of 10 seconds each, with congruent, and incongruent blocks, randomized across trials. Event timestamps marking the onset of each congruent and incongruent block were generated on PsychoPy and transmitted via Lab Streaming Layer (LSL) to Aurora Acquisition Software, where they were embedded as digital triggers with the fNIRS recording. Triggers were subsequently used to segment and average hemodynamic responses for each 10-second task block and corresponding 15 second rest period to enable average block analysis. All signal preprocessing steps, and handling of missing and noisy channels are described in Zondo et al. (2024a).

### Data analysis

***Behavioural performance***

Statistical analysis was performed using SPSS Statistics Version 27. First, the normality of the behavioural data was evaluated using the Shapiro-Wilk test. Then, the reaction time (in milliseconds) and accuracy (in total correct scores) data of the SCWT were analysed within groups and between groups (experimental vs control). Cohen’s *d* reported effects sizes.

***fNIRS***

**Figure 1.**
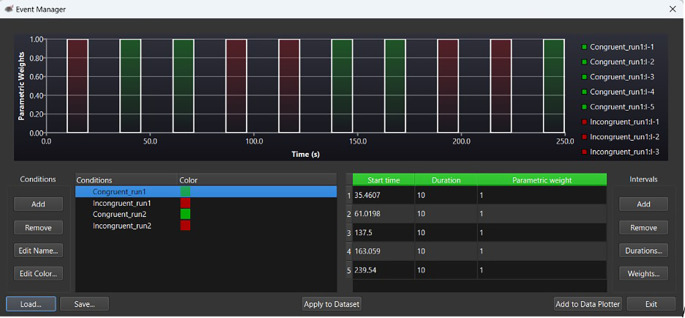
Event marker evaluating changes in oxygenated hemoglobin (ΔHbO), at pre- and post-test. *Note.* Green bars under ‘parameter weights’ indicate congruent trials. Red bars indicate incongruent trials. Within the ‘Conditions’ panel, *run1* indicates pretest HbO levels; *run2*, indicates post intervention HbO levels. For the above example, the congruent stimuli time course is indicated in green within the ‘Intervals’ (seconds) panel.

Using guidelines for the analysis of repeated measures in fNIRS (NIRx Medical Technologies), standardized beta coefficients were first derived using Satori. For each channel, pre-processing filters were applied, followed by convolving each stimulus (congruent, incongruent) to a hemodynamic response model. General Linear Models were then applied with the measured fNIRS (oxyHb and Hb) signals as dependent variables, and the convolved functions as independent variables. The beta values of these models were used as estimates for hemodynamic responses for the brain regions of interest. The sign and magnitude of each beta coefficient provided an indicator of the direction (positive/negative) and intensity of concentration changes in HbO (i.e., cortical activity) for each condition. Once all beta values were estimated, we then pattened our analysis following Khoe et al. (2020). As such, beta values representing HbO response activation were recorded at pre-training, and these served as a baseline for post-training hemodynamic response comparison. Primary outcomes for HbO activation were, therefore, changes in HbO, at post-training, minus those at pre-training (ΔHbO = (Post-intervention HbO) − (Pre-intervention HbO)). Concentration changes (ΔHbO) were calculated for congruent and incongruent trials for both groups. Figure 1 provides an example of an event marker to calculate ΔHbO at pre- and post-test within Satori fNIRS. No brain surface maps were used for the study. All optode placements were confirmed using the fOLD software ( Zimeo et al., 2018).

Changes in HbO (ΔHbO) within the experimental and control group were then represented by topographical maps of regions of the prefrontal cortex. Increase in brain hemodynamic response was represented in red. Increased oxygenated hemoglobin levels correlated with increased hemodynamic activation and cognitive effort. Light or dark blue represented hemodynamic deactivation and decreased brain activity. The Shapiro-Wilk Test evaluated normality of changes in oxygenated hemoglobin (ΔHbO), pre- and post-training. Differences in pre- and post-measures within each group were calculated using paired samples t-test. Between-group differences in ΔHbO were evaluated using an independent samples t-test. The Benjamini-Yekutieli method ( Benjamini & Yekutieli, 2001) adjusted for multiple comparisons.

## Results

### Behavioural data

***Within subjects group analyses***

The reaction time and accuracy scores of the SCWT at pre- and post-test, for the groups are presented in Table 2. For the experimental group, within-group analyses indicated no significant differences in reaction times when correctly identifying *congruent* trials ( *M *= -0.01; *SD* = 0.43, *t* (170) = - 0.45, *p* = 0.469, *d* = 0.43). Notably, although not significant, this group’s accuracy rates improved from 52% at pre-test to 61% post-test (p > 0.05). At pre-test, this group accurately identified 54.9% of incongruent trials, with a minimal improvement of 57% at post-test (p > 0.05). Notably, although there were increases in accuracy rates (%) at post-test, reaction times were significantly slower at post-test ( *M* = 1.24), compared to pre-test ( *M* = 1.09; *p* = 0.001, *d* = 0.6).

**Table 2.** Reaction time and accuracy % on the SCWT.

Variable	Exp M	Exp SD	Ctrl M	Ctrl SD	Statistic	p	Effect size
**Reaction Time (s) — Congruent**							
Pre	1.09	0.30	0.97	0.02	*t = 1.439*	*.176*	*d = 0.564*
Post	1.03	0.20	1.17	0.07	*t = −2.382*	*.031*	*d = 0.934*
**Reaction Time (s) — Incongruent**							
Pre	1.24	0.40	1.03	0.20	*t = 1.693*	*.108*	*d = 0.664*
Post	1.27	0.50	1.15	0.03	*t = 0.864*	*.405*	*d = 0.339*
**Accuracy (%) — Congruent**							
Pre	52.0%	—	47.8%	—	*z = 0.214*	*.830*	*h = 0.084*
Post	61.4%	—	39.1%	—	*z = 1.137*	*.256*	*h = 0.450*
**Accuracy (%) — Incongruent**							
Pre	54.0%	—	45.1%	—	*z = 0.454*	*.650*	*h = 0.178*
Post	57.0%	—	43.4%	—	*z = 0.693*	*.488*	*h = 0.273*

**Note.** Exp = Experimental group (n = 13); Ctrl = Control group (n = 13). Reaction time comparisons used Welch’s independent-samples t-tests; Cohen’s d reported as effect size. Accuracy comparisons used two-proportions z-tests; Cohen’s h reported as effect size. SD is not presented for accuracy (%) as values are summary proportions (—). Significance threshold: p < .05.

Within-groups analyses for the control group indicated an accuracy rate of 48% for congruent trials at pre-test and 39% at post-test. On average, participants indicated significantly slower reaction times (millisecond) at post-test ( *M* = 1.37; SD = 0.81) compared to pre-test ( *M* = 0.97) when correctly identifying congruent trials ( *M* = -0.33; *SD* = 0.91, *t* (108) = -3.85, *p* = 0.011, *d* = 0.91). At pre-test, participants accurately identified 45.1% of incongruent trials and 43.4% at post-test (p > 0.05). On average, participants’ reaction time was significantly slower at post-test ( *M* = 1.17; *SD* = 0.6) when identifying incongruent trials ( *M* = 0.11; *SD* = 0.47, *t* (134) = -2.85, *p* = 0.05, *d* = 0.47).

***Between subject group analyses***

Between group analyses indicated no significant differences between the group’s reaction times at pre- and post-test, when correctly identifying congruent trials ( *U* = 1175, *p* = 0.76). Similarly, no significant differences were found at pre- and post-test between the groups in terms of reaction times when accurately identifying incongruent trials ( *U* = 1200, *p* = 0.065).

### fNIRS Neuroimaging results

***PFC brain activation: Pre & Post intervention: Congruent trial***

**Figure 2.**
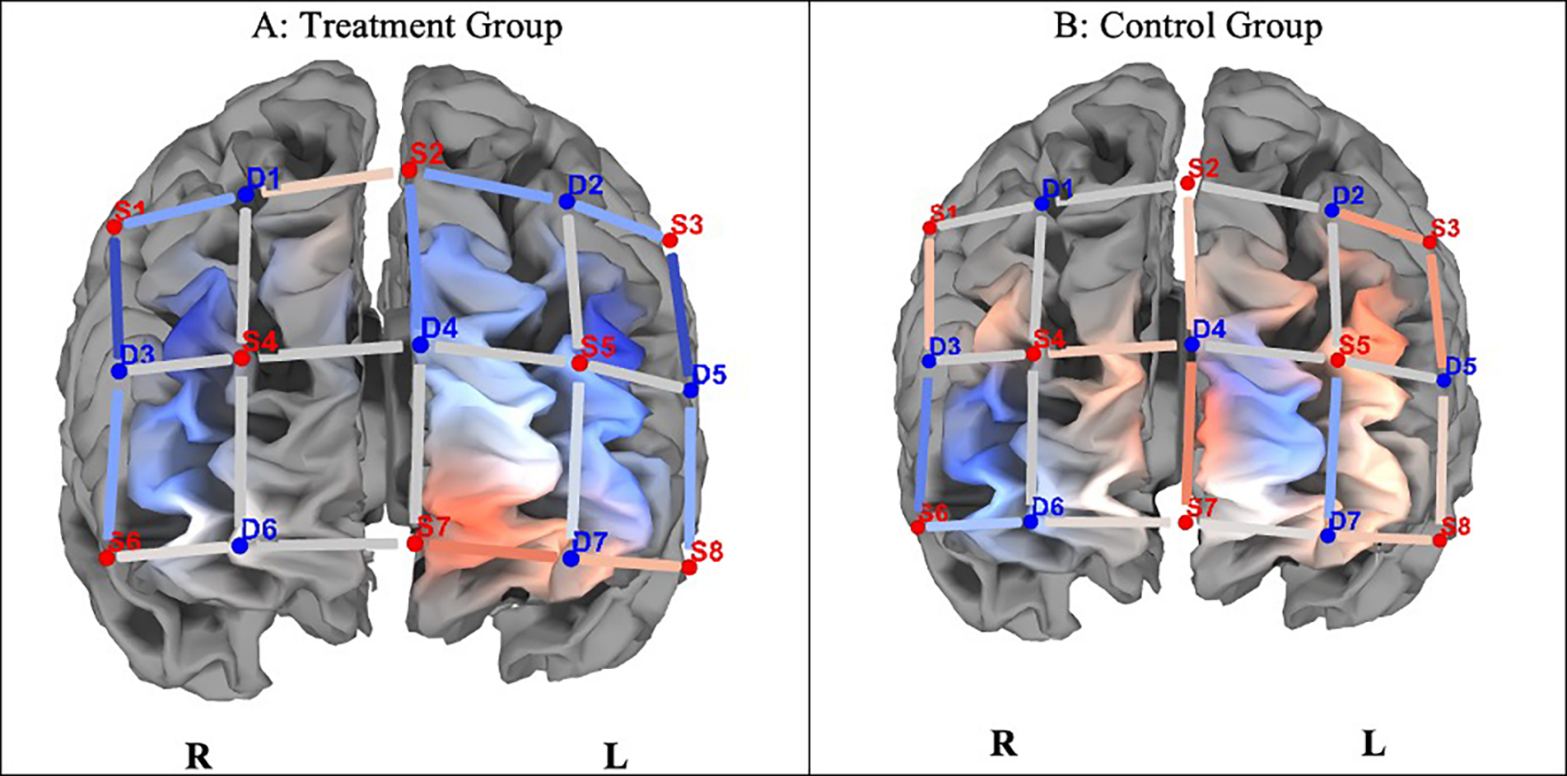
Changes in PFCA (ΔHbO) between the treatment and control groups on congruent trials. *Note.* **A**. The experimental group indicated lesser hemodynamic changes at pre- and post-test (ΔHbO). **B**. The control group shows greater hemodynamic changes on pre- and post-test congruent trials (ΔHbO), but they were not statistically significant ( *p* > 0.05). Concentration of colour blue denotes less HbO, and red, greater HbO.

Differences in brain activation are indicated by contrast t-statistics maps for HbO. We compared brain activation in the prefrontal cortex at pre- and post-test in relation to congruent and incongruent trials on the SCWT. Figure 2 indicates neuronal differences in ΔHbO between the groups during the congruent task. Although the control group indicated greater differences in HbO activation (ΔHbO), there were no significant differences (p > 0.05) between the groups on any of the optodes in the regions of interest.

***Prefrontal cortex activation: Pre- and Post-test: Incongruent trials***

**Figure 3.**
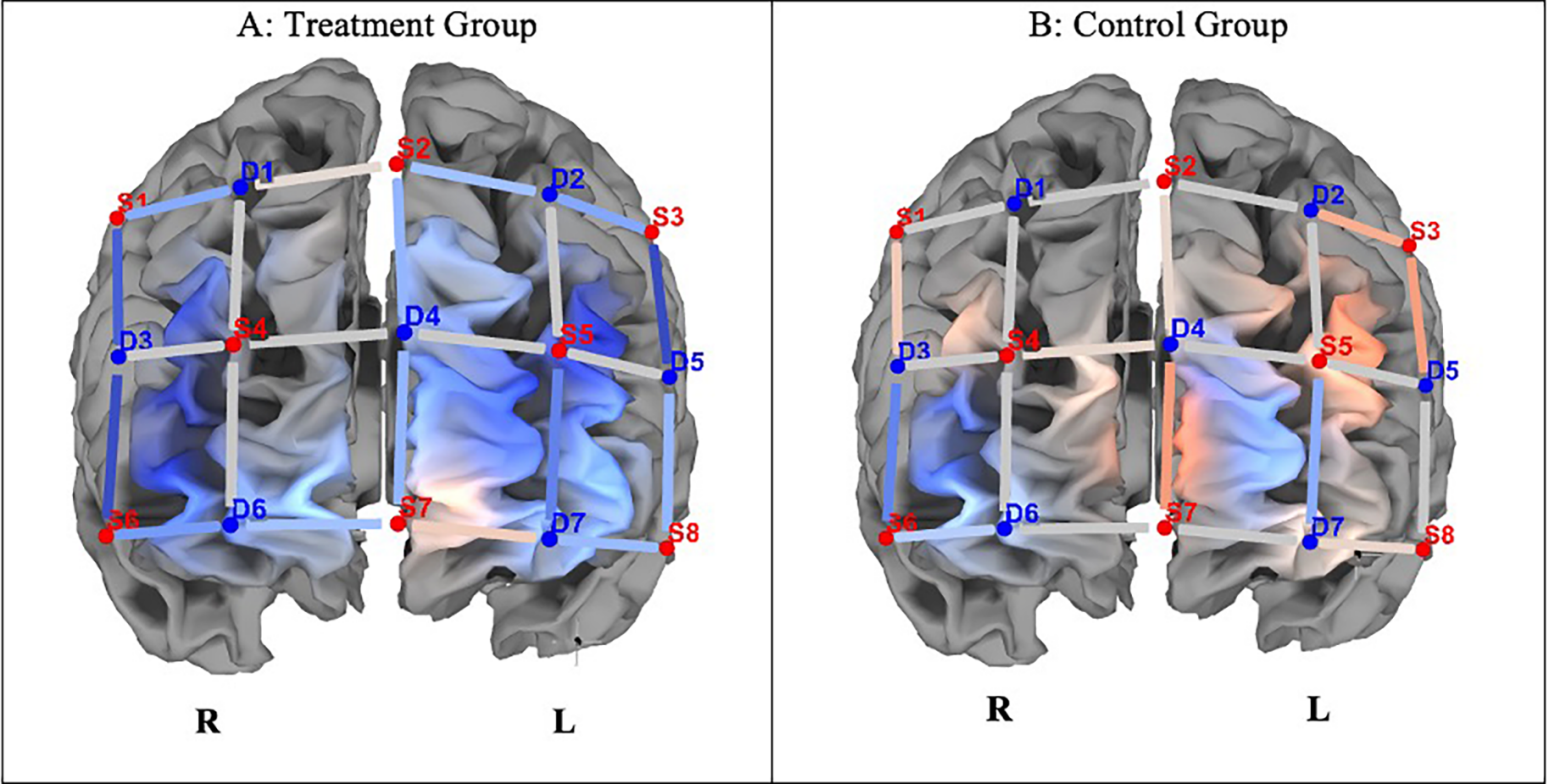
Changes in PFCA (ΔHbO) between the experimental and control groups on incongruent trials. *Note.* **A**. The experimental group indicated lesser hemodynamic changes at pre- and post-test (ΔHbO). **B**. The control group indicated greater hemodynamic changes in pre- and post-test incongruent trials (ΔHbO). Concentration of colour blue denotes less HbO, and red, greater HbO.

Figure 3 shows differences in ΔHbO between the groups. Independent t-tests revealed that completing incongruent trials resulted in significantly lower hemodynamic responses in the left and right dorsolateral prefrontal cortex (DLPF) and left frontopolar Area ^5^ in the experimental group compared to the control group (p < 0.05; see Table 3).

**Table 3.** Areas of significantly lower activation in the HIV treatment group in relation to completing incongruent trials.

Channel	Optode name	MNI position			BA	Anatomical region
		***x***	***y***	***z***		
CH 2	S1 - D3	46	38	24	45	Right pars triangularis Broca’s Area
					46	Right dorsolateral prefrontal cortex
CH 7	S3 - D5	-46	39	26	45	Left pars triangularis Broca’s Area
					46	Left dorsolateral prefrontal cortex
CH 14	S6 - D3	48	46	5	45	Right pars triangularis Broca’s Area
					46	Right dorsolateral prefrontal cortex
CH 18	S7 - D7	-12	67	0	10	Left frontopolar area
					11	Left Orbitofrontal area
CH 19	S8 - D5	-47	46	6	45	Left pars triangularis Broca’s area
					46	Left dorsolateral prefrontal cortex
CH 20	S8 - D7	-23	62	23	9	Left dorsolateral prefrontal cortex
					46	Left dorsolateral prefrontal cortex
					10	Left frontopolar Area

*Note.* S = Source; D = Detector; MNI = Montreal Neurological Institute; BA = Brodman Area.

## Discussion

Disturbance in neural cytoarchitecture following HIV-infection is associated with decreased cognitive function, particularly in attention and executive functions, such as working memory ( Hammond et al., 2019). Significantly, cART cannot reverse cognitive decline ( Gonzalez et al., 2020). Functional neuroimaging studies suggest individuals living with HIV typically show lower BOLD activation in the CEN on simple undemanding tasks, but greater BOLD activation as cognitive load increases, in both the CEN and compensatory brain regions (i.e., the parietal regions) ( Chang & Shukla, 2018). In individuals with neuroHIV, increased BOLD activation is typically a marker of neuronal inefficiency, as the cortex overcompensates and recruits associated neural networks in response to increasing cognitive demands ( Chang et al., 2004, 2013).

With adults, there is evidence that working memory training may result in greater cognitive proficiency in related tasks and is associated with decreased BOLD activation, symbolizing neural efficiency and task proficiency ( Chang et al., 2017). No empirical studies exist investigating changes in BOLD or hemodynamic responses in children or adolescents living in Sub-Saharan Africa, which continues to bear the burden of neuroHIV. Given this dearth, our study investigated whether customized attention remediation improved performance on the SCWT (a measure of attention and inhibitory control) and also whether any such cognitive outcomes were related to decreased hemodynamic response activation within the prefrontal cortex. Potential brain plasticity was investigated in our participants using optical neuroimaging in the form of fNIRS.

Although no significant behavioural differences were observed on the Stroop task, ALWH who received sustained attention training showed modest improvements in congruent trial accuracy, while the control group’s performance declined. Incongruent trial accuracy remained largely unaffected by training. Notably, significant between-group differences emerged in hemodynamic responses during incongruent trials, suggesting neural effects of the intervention despite limited behavioural impact.

With reference to neural effects, we investigated whether improvements on the SCWT correlated with decreased hemodynamic responses in the prefrontal cortex following the attention training. While the control group showed increased hemodynamic response activation on ΔHbO compared to the experimental group on congruent trials, these changes were statistically insignificant. Concerning incongruent trials, there were significant differences in ΔHbO between the groups, with the control group showing increased hemodynamic responses, and the experimental group indicating attenuation in hemodynamic responses, suggesting greater neural efficiency in the latter group in dedicated brain areas. Defined cortical regions of hemodynamic attenuation were localised to the frontopolar/anterior prefrontal area, dorsolateral PFC, the orbitofrontal region, and the pars triangularis Broca’s Area, when completing incongruent trials of the SCWT.

The SCWT interference task typically places significant cognitive load on cortical processes, resulting in hyperactivation of cortical regions, including the dorsal, orbitofrontal, medial prefrontal and ventrolateral prefrontal cortex ( Banich, 2019). Our findings suggest that attention training decreases hemodynamic responses in the dorsolateral prefrontal cortex, a key network node with the CEN. Since the dorsolateral prefrontal network is connected to the orbitofrontal cortex through association fibres, the concomitant decrease in hemodynamic responses in these regions may suggest that cognitive training could strengthen participants’ ability to inhibit automatic responses and enable better monitoring and adjustment of their response strategies (which improved their accuracy on incongruent trials of the SCWT) ( Spielberg et al., 2015).

The findings from our study corroborate those of Chang et al. (2017), who observed decreased BOLD activation in the dorsal and lateral prefrontal cortices following working memory intervention. In their study, decreased BOLD activation was accompanied by improved performance on the 2-back working memory task. Where our findings differ from those by Chang et al. (2017), is that we did not find concomitant increased scores on the SCWT at post-test.

Notwithstanding the absence of significant cognitive test score improvements following the attention training, decreased cortical activation in the frontopolar area, as observed in the experimental group, is an interesting finding. This area is implicated in maintaining alertness and subsequent retrieval of stored information ( Hogeveen et al., 2022). Due to projections of the frontopolar region to the anterior cingulate cortex, the latter which is implicated in neuroHIV ( Israel et al., 2019; Toich et al., 2017), decreased hemodynamic activation in Area 10 may suggests improved neural efficiency and cortical proficiency. Moreover, attenuation in hemodynamic responses may indicate reduced involvement of the ‘top-down attention network’ (CEN) during demanding cognitive tasks such as the SCWT ( Banich, 2019).

Importantly, we found decreased hemodynamic responses in the left pars triangularis (Broca’s Area) and the orbitofrontal area. Broca’s area is activated during the completion of the SCWT, especially in incongruent trials ( Wallentin et al., 2015). To this end, the incongruent trial of the SCWT places significant cognitive load on language processes that require identifying the ink colour of the stimulus, while inhibiting any conflict that arises with the actual word name (which is also a colour). In addition to correctly identifying incongruent stimuli, participants manipulated visual data (colour/word) and employed inhibitory control, in order not to associate a colour with a word (colour name), thus engaging the orbitofrontal cortex ( Rolls et al., 1996). Thus, a decrease in hemodynamic activation in this region suggests decreased requirement for ‘top-down’ attention processing, which was found following the training.

Although the experimental group showed cortical-level changes in hemodynamic activity, these neural effects were not accompanied by significant improvements in SCWT accuracy or reaction times compared to controls. This dissociation between neural and behavioural outcomes is consistent with prior findings, such as those reported in a systematic review by Brooks et al. (2020), which noted that neural adaptations—including changes in BOLD activation and functional connectivity—often precede observable cognitive gains. Such delays may be influenced by factors like training duration, neural plasticity, and intervention intensity. These findings suggest that early neural changes may reflect emerging plasticity, with behavioural improvements potentially requiring extended intervention periods to manifest. Several empirical studies support this view ( Powell & Redish, 2016; Tremblay et al., 1998).

### Limitations

Despite promising findings—namely, reduced prefrontal hemodynamic activation post-training and improved SCWT performance on incongruent trials—several limitations must be acknowledged. Due to the nature of the sample (vulnerable children living with HIV), the study experienced high attrition, which led to a reduced sample size, limiting statistical power, and pre-test group differences in age and sex may have influenced outcomes. Additionally, important moderators such as HIV genotype, age of cART initiation, viral load status, and individual neurobiological variability ( Brew & Garber, 2018), were not available, and hence not assessed, affecting further analysis. Moreover, we only investigated neural changes within a sample of adolescents living with HIV, and did not include negative controls, affecting comparison with typical, behavioural or hemodynamic patterns related to brain plasticity and neuronal development. Related to the above, neural changes were investigated within the cohort, one month after the cognitive training and further post-test follow ups (i.e., 6 months) would have added valuable data about whether any effects were sustained over time, or typical to negative controls. Moreover, the fNIRS protocol was limited in spatial coverage ( *Extended Data - Supplementary File*) and limited to HbO analysis and lacked short-separation channels to control for physiological noise ( Pfeifer et al., 2018). Future research could include a larger cortical coverage (i.e., fronto-parietal network), to investigate the effect of attention training on other cognitive modalities such as working memory. Lastly, one participant had a diagnosis of ADHD and was receiving stimulant medication which influences prefrontal hemodynamic responses ( Poliakova et al., 2023). Although this represents a single case, the inclusion of the participant in the group level analysis of HbO data, might have affected study findings.

## Conclusions

This appears to be the first study investigating hemodynamic responses related to attention training in pediatric neuroHIV. Observed neural attenuation suggests emerging neuroplasticity, supporting the potential of attention training—alongside cART—to enhance cognitive outcomes in children and adolescents affected by neuroHIV. Translational research can further adopt single case experimental designs (SCEDs), to enable delivery of intensive training sessions (30–40 mins), with fewer participants (6-8), to reduce study attrition. Moreover, instead of computer - based interventions, to reduce study costs, translational research can implement pencil and paper interventions, whereby lay counselors and community health practitioners can be trained to administer the intervention. Notwithstanding, findings from the study demonstrate the efficacy of brain intervention protocols and emergent neuroplasticity, within a cohort of adolescents living with HIV in Sub-Saharan Africa settings.

## Data Availability

All data underlying the results are available as part of the article and no additional source data are required. Extended data can be found on the below DOI. Zenodo: HIV Cognitive Rehabilitation BRIEF and NEPSY Data. https://doi.org/10.5281/zenodo.19022027 This project contains the following extended data:
- Dataset - Supplementary data - Footnote information
