# Supplementary material for: Shining Light into Adolescent HIV Neuroplasticity: A Study of the Prefrontal Cortex Using Functional Near Infrared Spectrometry
Source: F1000Res. 2026 Apr 24;14:1000. Originally published 2025 Sep 29. [Version 2] doi: 10.12688/f1000research.169799.2 (PMC13218407; doi:10.12688/f1000research.169799.2)
Supplement: Peer review history

# Reviewer report: Reviewer response for version 2

**DOI:** 10.5256/f1000research.197457.r478731

**Published:** 2026-05-19

**Authors:** Nerrolyn Ramstrand

**Affiliations:** 1 Jönköping University, Jönköping, Sweden

Many thanks to the authors for addressing my initial comments in relation to this manuscript.  I have no additional comments to make and wish the author team all the best.

Is the work clearly and accurately presented and does it cite the current literature?

Partly

If applicable, is the statistical analysis and its interpretation appropriate?

Partly

Are all the source data underlying the results available to ensure full reproducibility?

Partly

Is the study design appropriate and is the work technically sound?

Yes

Are the conclusions drawn adequately supported by the results?

Yes

Are sufficient details of methods and analysis provided to allow replication by others?

No

Reviewer Expertise:

fNIRS, cognitive load, prosthetics and orthotics

I confirm that I have read this submission and believe that I have an appropriate level of expertise to confirm that it is of an acceptable scientific standard.

# Reviewer report: Reviewer response for version 1

**DOI:** 10.5256/f1000research.187175.r441630

**Published:** 2026-01-06

**Authors:** Taylor Fitzpatrick-Schmidt

**Affiliations:** 1 Louisiana State University Health Sciences Center, New Orleans, Louisiana, USA

This study investigated the impact of attention training on cognitive performance using the Stroop Color Word Test and cortical hemodynamic responses, a proxy for neural activation, using functional near-infrared spectroscopy (fNIRS) in HIV-positive adolescents. Overall, the manuscript is well-written and has several strengths, including its focus on an understudied population and the integration of both behavioral and neuroimaging measures. I recommend addressing the following points within the scope of this article.

**Abstract**

 Overall, the abstract clearly conveys the main findings and take-home message of the study. It could be strengthened by briefly reporting the behavioral results in the Results section, even though the results are not significant, and adding a short sentence to provide interpretation of the changes in oxygenated hemoglobin as done in the discussion (e.g., greater neural efficiency), as described in the Discussion section of the main body. This would help more clearly link the Results and Conclusions sections.

**Introduction**

 The introduction effectively establishes the importance of studying interventions targeting cognitive deficits in HIV-positive children and adolescents and provides a solid overview of prior neuroimaging work in this population. This section could be streamlined by reducing methodological detail from previous studies and by placing greater emphasis on the rationale for focusing on attention at the targeted cognitive domain and the prefrontal cortex as the primary region of interest.  

**Methods and Results**

 The Methods and Results sections are generally well-organized. The following points may further strengthen these sections:

1. If available, include information on age at HIV diagnosis and indicators of disease severity (e.g., CD4 count, viral load), as these variables may influence baseline cognitive task performance.  
2. Consider conducting correlational analyses to investigate associations between behavioral performance and neuroimaging results.
3. Given baseline differences between treatment and control groups (especially age and sex), consider adjusting analyses for relevant covariate, if the sample size permits.
4. Including p-values in Table 2 (even for non-significant results) would improve transparency.

**Discussion**

 Overall, the discussion effectively integrates the results of this study with the existing literature. A couple points to consider:

1. Consider introducing study limitations earlier in the Discussion rather than in the Conclusions. In addition to those already noted, the inclusion of only HIV-positive participants should be acknowledged as a limitation.
2. Expanding on how these findings inform future research, such as examining other cognitive domains (e.g., memory), would further strengthen this section.

Is the work clearly and accurately presented and does it cite the current literature?

Partly

If applicable, is the statistical analysis and its interpretation appropriate?

Partly

Are all the source data underlying the results available to ensure full reproducibility?

Partly

Is the study design appropriate and is the work technically sound?

Yes

Are the conclusions drawn adequately supported by the results?

Yes

Are sufficient details of methods and analysis provided to allow replication by others?

Partly

Reviewer Expertise:

HIV-associated neurocognitive disorders, HIV-associated neuropathy, alcohol use disorder

I confirm that I have read this submission and believe that I have an appropriate level of expertise to confirm that it is of an acceptable scientific standard, however I have significant reservations, as outlined above.

# Response

**Published:** 2026-03-09

**Authors:** Sizwe Zondo

This study investigated the impact of attention training on cognitive performance using the Stroop Color Word Test and cortical hemodynamic responses, a proxy for neural activation, using functional near-infrared spectroscopy (fNIRS) in HIV-positive adolescents. Overall, the manuscript is well-written and has several strengths, including its focus on an understudied population and the integration of both behavioral and neuroimaging measures. I recommend addressing the following points within the scope of this article.

**Abstract**

 Overall, the abstract clearly conveys the main findings and take-home message of the study. It could be strengthened by briefly reporting the behavioral results in the Results section, even though the results are not significant, and adding a short sentence to provide interpretation of the changes in oxygenated hemoglobin as done in the discussion (e.g., greater neural efficiency), as described in the Discussion section of the main body. This would help more clearly link the Results and Conclusions sections.

**Response:**

 Thank you for your comment. The abstract has since been revised to explicitly report the behavioural results. Additionally, a brief interpretive statement has been added to contextualize the observed changes in oxygenated haemoglobin levels.

**Introduction**

 The introduction effectively establishes the importance of studying interventions targeting cognitive deficits in HIV-positive children and adolescents and provides a solid overview of prior neuroimaging work in this population. This section could be streamlined by reducing methodological detail from previous studies and by placing greater emphasis on the rationale for focusing on attention at the targeted cognitive domain and the prefrontal cortex as the primary region of interest.  

**Response:**

 It is acknowledged that the focus on attention is not emphasized – to rectify this anomaly the manuscript has added a section on the construct of attention and linked this construct to prefrontal lobe anatomy and the regions of interest of the study.  

**Methods and Results**

 The Methods and Results sections are generally well-organized. The following points may further strengthen these sections:

1. If available, include information on age at HIV diagnosis and indicators of disease severity (e.g., CD4 count, viral load), as these variables may influence baseline cognitive task performance.  

**Response:**

 Thank you for this comment. Unfortunately, at the time of data collection, this critical medical data was not available to the researchers. The research was executed at data sites that care for children living with HVI and not at tertiary clinical sites where the data might be easily available. The sites were unfortunately unable to provide this key biomarker data, except for medication other relevant data.  

  

1. Consider conducting correlational analyses to investigate associations between behavioral performance and neuroimaging results.

**Response:**

 We thank the reviewer for this constructive suggestion. We agree that examining associations between behavioural performance and hemodynamic responses would further clarify the functional significance of the observed neuroimaging findings. While the current study was primarily designed to evaluate group-level differences, correlational analyses examining brain–behaviour relationships are currently underway as part of an ongoing extension of the study dataset. These analyses will form the basis of a subsequent manuscript submission focusing on neurocognitive–hemodynamic coupling. The article we are currently working will investigate associations, as well as (b) efficiency index analysis, and (c) implement moderation models to investigate cognition (all attention and NEPSY measures) by group and hemodynamic responses

1. Given baseline differences between treatment and control groups (especially age and sex), consider adjusting analyses for relevant covariate, if the sample size permits.

**Response:**

 Thank you very much for this suggestion. This implementation is being extended to form part of the above noted article (the power analysis is weak at this stage, due to a small sample size).

  

1. Including p-values in Table 2 (even for non-significant results) would improve transparency.

**Response:**

 We have since revised the Table 2 with the necessary statistics for replicability and transparency.

**Discussion**

 Overall, the discussion effectively integrates the results of this study with the existing literature. A couple points to consider:

1. Consider introducing study limitations earlier in the Discussion rather than in the Conclusions. In addition to those already noted, the inclusion of only HIV-positive participants should be acknowledged as a limitation.

**Response:**

 Thank you for the suggestion, we have since created a Limitations section to the manuscript and noted the above limitation.

 Expanding on how these findings inform future research, such as examining other cognitive domains (e.g., memory), would further strengthen this section.

**Response:**

 We have incorporated this suggestion and the importance of translational research in the Discussion section.

 We thank the reviewer for all the above observations which we believe have strengthened the academic integrity of this work.

# Reviewer report: Reviewer response for version 1

**DOI:** 10.5256/f1000research.187175.r425390

**Published:** 2026-01-05

**Authors:** Kaylee Van Wyhe

**Affiliations:** 1 Stellenbosch University, Stellenbosch, Western Cape, South Africa

This manuscript presents an innovative and timely investigation of prefrontal cortical hemodynamics in adolescents living with HIV using functional near-infrared spectroscopy (fNIRS). The authors examine neural changes associated with an attention-training intervention and pair these findings with behavioural outcomes on the Stroop Colour–Word Test (SCWT). The topic is highly relevant to the field of pediatric neuroHIV, and the study offers promising insights into neuroplasticity in adolescents. Overall, I am supportive of the manuscript and believe it makes a meaningful contribution. My comments below are intended to strengthen clarity, contextual accuracy, and methodological transparency.

**Major Comments**

**1. Title and overall framing**

 The title is engaging, well constructed, and suitably reflects the study’s scope. I note that its clarity and precision support the paper’s impact and readability.

**2. Appropriateness of fNIRS**

 The authors’ decision to use fNIRS is well justified, especially given its portability, tolerance for movement, and ability to measure both oxygenated and deoxygenated hemoglobin, all suited to pediatric and adolescent populations. This strengthens the methodological rationale.

**Introduction**

**3. Update epidemiological statistics**

 The introduction references UNAIDS 2019 estimates. More recent estimates are available (e.g., 2022 and 2024 updates). Revising these statistics will ensure accuracy and reinforce the contemporary relevance of the study.

**4. ART toxicity statement needs clarification**

 The introduction states that combination ART is “toxic,” referencing Yuan & Kaul (2019). This framing is historically accurate for older regimens but does not reflect current treatment realities. Contemporary cART regimens are significantly safer, well-tolerated, and overwhelmingly beneficial, improving survival, preventing disease progression, and completely preventing HIV transmission when virally suppressed. I strongly recommend clarifying this distinction to avoid reinforcing outdated or stigmatizing narratives. A brief note highlighting reduced toxicity in newer regimens would enhance scientific accuracy.

**5. Outdated literature on cognitive deficits**

 The paper relies on Milligan & Cockcroft (2017) to support statements about executive functioning deficits. Given the rapid advancements in pediatric neuroHIV research, it would strengthen the introduction to reference more recent evidence.

**6. Missing behavioural interpretation from Chang et al. (2001)**

 The introduction uses Chang et al. (2001) to discuss altered brain activation patterns but omits a key behavioural context: whether hyperactivation was associated with comparable accuracy but slower processing speed. Including the behavioural outcomes would give a more complete foundation for discussing BOLD-like hemodynamic responses and compensatory hyperactivation in HIV.

**7. Language and terminology regarding HIV**

 The manuscript frequently uses “HIV+.” It is recommended to adopt people-first, non-stigmatizing terminology, such as “adolescents living with HIV (ALWH).” Applying consistent, person-first language will align the manuscript with current global guidelines.

**Methods**

**8. Participant viral suppression status**

 The methods section states that all participants were on cART but does not indicate whether they were virally suppressed. Viral load status is a critical clinical characteristic that can meaningfully influence neurocognitive functioning. Please clarify suppression status if available.

**9. Age categorisation using WHO groupings**

 Participants are divided into 0–13 and 14–18 age groups using WHO categories. While I understand the rationale, this results in a wide “0–13” group containing 11 participants across a period of rapid neurodevelopment (childhood → early adolescence). Given the mean age of 17 years, it would be scientifically more meaningful to report exact ages or narrower developmental bands. Prefrontal cortex maturation differs substantially between a 7-year-old, a 12-year-old, and a 17-year-old. Reporting mean age, SD, and a narrower range would improve interpretability.

**10. Education levels**

 Rather than reporting “primary vs secondary schooling,” providing duration of education (e.g., mean years of schooling completed) would offer greater developmental and socio-demographic insight.

**11. Outlier participant**

 One participant was excluded for responding to fewer than 38% of trials and pressing the same key for all SCWT conditions. It would be useful to report:

- the participant’s age
- whether they were virally suppressed
- whether their behavioural pattern suggested non-engagement, motor difficulty, or attentional impairment

This information strengthens transparency around exclusion criteria and may offer insight into neurodevelopmental or behavioural variability within the cohort.

**Discussion and Conclusion**

**12. Real-world implications and applicability**

 The discussion is strong, but I encourage the authors to briefly comment on the feasibility and clinical implications of implementing attention training in real-world settings. For example:

- approximate duration of training per session
- potential cost or resource requirements
- suitability for low-resource or community-based environments
- whether the intervention can be delivered by non-specialists

Even a brief, context-aware comment would enhance translational value.

**13. Excellent summary of limitations**

 The limitations section is thorough, well-considered, and enhances credibility. I agree that factors such as HIV genotype, age of cART initiation, neurobiological heterogeneity, and short-separation channel absence are meaningful limitations. The acknowledgement of sample attrition in vulnerable populations is also appropriate.

**Concluding Remarks**

 This is an important and innovative study that meaningfully advances our understanding of neuroplasticity in adolescents living with HIV. The combination of fNIRS with behavioural assessment is commendable, and the findings offer promising indications that attention training may serve as a valuable adjunct to cART. Addressing the points above, primarily related to updated epidemiological data, clarifying ART statements, refining participant descriptions, and strengthening real-world context, will improve clarity and enhance the manuscript’s impact. I am supportive of publication following these revisions.

Is the work clearly and accurately presented and does it cite the current literature?

Partly

If applicable, is the statistical analysis and its interpretation appropriate?

Yes

Are all the source data underlying the results available to ensure full reproducibility?

Partly

Is the study design appropriate and is the work technically sound?

Yes

Are the conclusions drawn adequately supported by the results?

Yes

Are sufficient details of methods and analysis provided to allow replication by others?

Partly

Reviewer Expertise:

Pediatric and adolescent HIV neuropsychology; developmental cognitive neuroscience; neuropsychological assessment and test adaptation in African populations; longitudinal neurodevelopmental research

I confirm that I have read this submission and believe that I have an appropriate level of expertise to confirm that it is of an acceptable scientific standard, however I have significant reservations, as outlined above.

# Response

**Published:** 2026-03-09

**Authors:** Sizwe Zondo

This manuscript presents an innovative and timely investigation of prefrontal cortical hemodynamics in adolescents living with HIV using functional near-infrared spectroscopy (fNIRS). The authors examine neural changes associated with an attention-training intervention and pair these findings with behavioural outcomes on the Stroop Colour–Word Test (SCWT). The topic is highly relevant to the field of pediatric neuroHIV, and the study offers promising insights into neuroplasticity in adolescents. Overall, I am supportive of the manuscript and believe it makes a meaningful contribution. My comments below are intended to strengthen clarity, contextual accuracy, and methodological transparency.

**Major Comments**

  

1. **Title and overall framing**
  The title is engaging, well constructed, and suitably reflects the study’s scope. I note that its clarity and precision support the paper’s impact and readability.

**Response:**

 We thank the reviewer for this positive comment.

**2. Appropriateness of fNIRS**

 The authors’ decision to use fNIRS is well justified, especially given its portability, tolerance for movement, and ability to measure both oxygenated and deoxygenated hemoglobin, all suited to pediatric and adolescent populations. This strengthens the methodological rationale.

**Introduction**

**3. Update epidemiological statistics**

 The introduction references UNAIDS 2019 estimates. More recent estimates are available (e.g., 2022 and 2024 updates). Revising these statistics will ensure accuracy and reinforce the contemporary relevance of the study.

**Response:**

 We have since updated the epidemiological data – and included the latest reference from WHO / UNAIDS (2024).

**4. ART toxicity statement needs clarification**

 The introduction states that combination ART is “toxic,” referencing Yuan & Kaul (2019). This framing is historically accurate for older regimens but does not reflect current treatment realities. Contemporary cART regimens are significantly safer, well-tolerated, and overwhelmingly beneficial, improving survival, preventing disease progression, and completely preventing HIV transmission when virally suppressed. I strongly recommend clarifying this distinction to avoid reinforcing outdated or stigmatizing narratives. A brief note highlighting reduced toxicity in newer regimens would enhance scientific accuracy.

**Response:**

 Thank you for this important point, we have since rephrased the section and subsequently included a note detailing reduced neurotoxicity of current cART regimens.

**5. Outdated literature on cognitive deficits**

 The paper relies on Milligan & Cockcroft (2017) to support statements about executive functioning deficits. Given the rapid advancements in pediatric neuroHIV research, it would strengthen the introduction to reference more recent evidence.

**Response:**

 In addition to the above key reference, we have updated the section by including a meta-analysis reference (2024) that indicates the persistence of cognitive deficits in this group.

**6. Missing behavioural interpretation from Chang et al. (2001)**

 The introduction uses Chang et al. (2001) to discuss altered brain activation patterns but omits a key behavioural context: whether hyperactivation was associated with comparable accuracy but slower processing speed. Including the behavioural outcomes would give a more complete foundation for discussing BOLD-like hemodynamic responses and compensatory hyperactivation in HIV.

**Response:**

 Thank you for this observation – we have since included a section expanding on the above, by highlighting accuracy levels as noted by the authors. 

**7. Language and terminology regarding HIV**

 The manuscript frequently uses “HIV+.” It is recommended to adopt people-first, non-stigmatizing terminology, such as “adolescents living with HIV (ALWH).” Applying consistent, person-first language will align the manuscript with current global guidelines.

**Response:**

 This observation is welcome and has been adopted into the manuscript when speaking in general terms pertaining to individuals living with HIV. However, the term ‘HIV+’ is retained for easier reporting and consistency within the manuscript, so as not to confuse ‘adults’ and ‘adolescents’ living with HIV.

**Methods**

**8. Participant viral suppression status**

 The methods section states that all participants were on cART but does not indicate whether they were virally suppressed. Viral load status is a critical clinical characteristic that can meaningfully influence neurocognitive functioning. Please clarify suppression status if available.

**Response:**

 We thank the reviewer for this important point. Data for the study was collected at children’s homes – with one nurse available at one of the homes. As noted in the discussion section, while all participants were receiving combination antiretroviral therapy, concomitant viral load measurement was not available on the children’s medical records and had to be directly sourced from the provincial hospital. The absence of viral load data is noted in the Limitations and Discussion section. We further agree that viral suppression status is clinically important, and an influential factor in neurocognitive functioning. This key data variable will be collected on the Phase 2 of our study.

**9. Age categorisation using WHO groupings**

 Participants are divided into 0–13 and 14–18 age groups using WHO categories. While I understand the rationale, this results in a wide “0–13” group containing 11 participants across a period of rapid neurodevelopment (childhood → early adolescence). Given the mean age of 17 years, it would be scientifically more meaningful to report exact ages or narrower developmental bands. Prefrontal cortex maturation differs substantially between a 7-year-old, a 12-year-old, and a 17-year-old. Reporting mean age, SD, and a narrower range would improve interpretability.

**Response:**

 This point is well taken, and a limitation of the study. The authors acknowledge that recategorization of the age variable, although expedient, would mean reanalysis of imaging data. We will however seek to take this consideration in future publications emerging from the current work.   

**10. Education levels**

 Rather than reporting “primary vs secondary schooling,” providing duration of education (e.g., mean years of schooling completed) would offer greater developmental and socio-demographic insight.

**Response:** 

 This is a much-appreciated observation. From the data collected at the HIV shelters, we could not ascertain the mean years of schooling completed; this data was either not available, or not forthcoming, as its availability could indirectly affect donor funding for certain populations of children. Nonetheless, the authors acknowledge the implication of this oversight for developmental and socio-demographic interpretability.

**11. Outlier participant**

 One participant was excluded for responding to fewer than 38% of trials and pressing the same key for all SCWT conditions. It would be useful to report:

- the participant’s age
- whether they were virally suppressed
- whether their behavioural pattern suggested non-engagement, motor difficulty, or attentional impairment

This information strengthens transparency around exclusion criteria and may offer insight into neurodevelopmental or behavioural variability within the cohort.

**Response:**

 Thank you for this observation. The relevant information has been incorporated into the manuscript. As indicated on the manuscript revisions, no data was available pertaining to viral suppression (CD4+ count), for research participants.

**Discussion and Conclusion**

**12. Real-world implications and applicability**

 The discussion is strong, but I encourage the authors to briefly comment on the feasibility and clinical implications of implementing attention training in real-world settings. For example:

- approximate duration of training per session
- potential cost or resource requirements
- suitability for low-resource or community-based environments
- whether the intervention can be delivered by non-specialists

Even a brief, context-aware comment would enhance translational value.

**Response:**

 We thank the reviewer for this suggestion. The Discussion section has been revised to include a brief comment on the feasibility and clinical implications of implementing attention training in real-world settings such as where the study was executed. Specifically, we comment on approximate sessions, their duration, and the use of pencil paper cognitive interventions for potential rehabilitation delivery by non-specialist facilitators.

**13. Excellent summary of limitations**

 The limitations section is thorough, well-considered, and enhances credibility. I agree that factors such as HIV genotype, age of cART initiation, neurobiological heterogeneity, and short-separation channel absence are meaningful limitations. The acknowledgement of sample attrition in vulnerable populations is also appropriate.

**Response:**

 We thank the reviewer for this helpful comment.

**Concluding Remarks**

 This is an important and innovative study that meaningfully advances our understanding of neuroplasticity in adolescents living with HIV. The combination of fNIRS with behavioural assessment is commendable, and the findings offer promising indications that attention training may serve as a valuable adjunct to cART. Addressing the points above, primarily related to updated epidemiological data, clarifying ART statements, refining participant descriptions, and strengthening real-world context, will improve clarity and enhance the manuscript’s impact. I am supportive of publication following these revisions.

**Response:**

 We thank the reviewer for all the above comments, and other observations to strengthen the academic integrity of this work.

# Reviewer report: Reviewer response for version 1

**DOI:** 10.5256/f1000research.187175.r425396

**Published:** 2025-11-05

**Authors:** Nerrolyn Ramstrand

**Affiliations:** 1 Jönköping University, Jönköping, Sweden

Thank you for the opportunity to review this before-and-after study investigating the effects of an attention training intervention on Stroop Colour Word Test (SCWT) performance and cognitive load (fNIRS). This research addresses a highly relevant and timely topic, particularly in paediatric populations living with HIV, who are at increased risk for neurocognitive deficits.

 The manuscript is well written, and the study design is appropriate for the stated aims. To support replication and enhance transparency, I recommend clarifying several issues in the methods section. My comments on these and other elements of the manuscript are outlined below.

**Introduction**

 The introduction provides a clear and compelling overview of the challenges faced by children and adolescents living with HIV, particularly regarding neurocognitive functioning. The authors are to be commended for effectively motivating the need for rehabilitation interventions targeting cognitive decline in this vulnerable population.

**Methods**

 While the methods section is generally well structured, additional detail and clarification would strengthen the manuscript:

**Participant Inclusion Criteria** - The inclusion of a participant with ADHD warrants further justification. One child was reportedly on ADHD medication, which may influence neural activity. Prior research has shown that children with ADHD exhibit distinct patterns of brain activation compared to neurotypical peers (e.g., Cui et al., 2024), and that pharmacological treatment can increase HbO concentrations in the prefrontal cortex (e.g., Poliakova et al., 2023). The potential confounding effects of this should be addressed.

**Measurement Instruments** - Although the measures are outlined in the published study protocol, readers would benefit from additional detail in the manuscript itself. Specifically:

- Please include information on the reliability and validity of the instruments used.
- Clarify the administration of the SCWT. According to the protocol, 10 blocks of 10 seconds were completed—this should be explicitly stated.
- Were congruent and incongruent blocks randomized?
- How were timestamps used to mark the start and end of each block? This is critical for understanding the block averaging process in the fNIRS analysis.

**fNIRS Data Processing and reporting** - The methods mention that deoxygenated hemoglobin (HbR) was captured, yet only oxygenated hemoglobin (HbO) results are reported. Please clarify the rationale for this choice. Additionally, the data analysis section should describe:

- Signal preprocessing steps
- Handling of missing or noisy channel data, if applicable

**Optode Placement and Channel Analysis** - The description of fNIRS optode placement is vague. Based on the figures, it appears that 15 optodes (8 sources and 7 detectors) were positioned over:

- Prefrontal and premotor areas (e.g., S1- S4, D1–D4)
- Parietal regions (e.g., S5–S8, D6-D7)
- Interhemispheric regions (e.g., D4, D6) Please clarify which channels were analyzed and whether regions of interest (ROIs) were defined. Also, describe how data blocks were averaged for statistical analysis.

**Results**

 The results section is generally well presented. However, the following improvements are suggested:

- Include significance values and effect sizes in Table 2 to enhance interpretability.
- Figures 2 and 3 do not clearly depict optode placement relative to the brain surface. Consider revising these to more accurately reflect spatial positioning.
- Table 3 raises questions about the anatomical labeling of optodes. For example:
  - Optodes S1–D3 and S6–D3 are used to represent the right dorsolateral prefrontal cortex (dlPFC), appearing anteriorly on the right side.
  - Optodes S8–D5 and S8–D7 are used for the left dlPFC but appear posteriorly on the left side. This discrepancy should be clarified.
- The manuscript does not mention any bad channels. Please confirm whether any channels were excluded due to poor signal quality.

**Discussion**

 The discussion is well articulated, but I encourage the authors to elaborate on the clinical implications of their findings. For instance, the emergence of significant between-group differences in hemodynamic responses during incongruent trials—despite limited behavioral effects is noteworthy and merits further exploration.

 Additionally, the manuscript lacks a dedicated limitations section. Currently, limitations are briefly mentioned in the conclusion. I recommend restructuring the discussion to include a clear and comprehensive limitations section, followed by a conclusion that synthesizes key findings without introducing new concerns.

Is the work clearly and accurately presented and does it cite the current literature?

Partly

If applicable, is the statistical analysis and its interpretation appropriate?

Partly

Are all the source data underlying the results available to ensure full reproducibility?

Partly

Is the study design appropriate and is the work technically sound?

Yes

Are the conclusions drawn adequately supported by the results?

Yes

Are sufficient details of methods and analysis provided to allow replication by others?

No

Reviewer Expertise:

fNIRS, cognitive load, prosthetics and orthotics

I confirm that I have read this submission and believe that I have an appropriate level of expertise to confirm that it is of an acceptable scientific standard, however I have significant reservations, as outlined above.

# Response

**Published:** 2026-03-09

**Authors:** Sizwe Zondo

Thank you for the opportunity to review this before-and-after study investigating the effects of an attention training intervention on Stroop Colour Word Test (SCWT) performance and cognitive load (fNIRS). This research addresses a highly relevant and timely topic, particularly in paediatric populations living with HIV, who are at increased risk for neurocognitive deficits.

 The manuscript is well written, and the study design is appropriate for the stated aims. To support replication and enhance transparency, I recommend clarifying several issues in the methods section. My comments on these and other elements of the manuscript are outlined below.

**Introduction**

 The introduction provides a clear and compelling overview of the challenges faced by children and adolescents living with HIV, particularly regarding neurocognitive functioning. The authors are to be commended for effectively motivating the need for rehabilitation interventions targeting cognitive decline in this vulnerable population.

**Response:**

 We thank the reviewer for this positive point.

**Methods**

 While the methods section is generally well structured, additional detail and clarification would strengthen the manuscript:

**Participant Inclusion Criteria** - The inclusion of a participant with ADHD warrants further justification. One child was reportedly on ADHD medication, which may influence neural activity. Prior research has shown that children with ADHD exhibit distinct patterns of brain activation compared to neurotypical peers (e.g., Cui et al., 2024), and that pharmacological treatment can increase HbO concentrations in the prefrontal cortex (e.g., Poliakova et al., 2023). The potential confounding effects of this should be addressed.

**Response:**

 We thank the reviewer for raising this important point. The primary inclusion criterion for the study was children and adolescents living with HIV. ADHD was not a target condition but was present as a comorbid diagnosis in one participant, reflecting clinical heterogeneity often observed in paediatric and adolescent HIV populations. While we acknowledge that ADHD and stimulant medication may influence prefrontal hemodynamic responses, only one participant in the sample had a reported ADHD diagnosis and was receiving pharmacological treatment. Given the small proportion of the sample represented by this case, it is unlikely that this individual meaningfully influenced the overall pattern of results. Nevertheless, we have clarified this in the Methods section and acknowledged the potential confounding influence in the Limitations section.

**Measurement Instruments** - Although the measures are outlined in the published study protocol, readers would benefit from additional detail in the manuscript itself. Specifically:

- Please include information on the reliability and validity of the instruments used.

**Response:**

 The validity of the SCTW, NEPSY, and BRIEF for South African, and African adolescent populations has since been included in the Methods section.

- Clarify the administration of the SCWT. According to the protocol, 10 blocks of 10 seconds were completed—this should be explicitly stated.

**Response:**

 The information was reported in the cited publication but has since been included in the revised manuscript under the Methods section.

- Were congruent and incongruent blocks randomized?

**Response:**

 Yes, there was randomization of blocks as per Schroeter et al., 2002. This information has been included in the revised manuscript.

- How were timestamps used to mark the start and end of each block? This is critical for understanding the block averaging process in the fNIRS analysis.

**Response:**

 We thank the reviewer for this point. Briefly, task timestamps were generated within PsychoPy and transmitted in real time to the Aurora Acquisition Software (NIRx) via Lab Streaming Layer (LSL). Specifically, digital event markers corresponding to the onset of each congruent and incongruent block were relayed to LSL stream at block initiation. These markers were embedded as time-locked trigger channels within the fNIRS recording. Markers were time-locked with each 10-second task block accompanied by an intervening 15-second rest period, allowing for block-averaging analysis in Satori fNIRS. We have clarified this procedure in the Methods section to improve transparency regarding the block-averaging process. As noted in the initial publication, our SCWT was adapted from Schroeter et al., 2002.

**fNIRS Data Processing and reporting** - The methods mention that deoxygenated hemoglobin (HbR) was captured, yet only oxygenated hemoglobin (HbO) results are reported. Please clarify the rationale for this choice. Additionally, the data analysis section should describe:

- Signal preprocessing steps
- Handling of missing or noisy channel data, if applicable

**Response:**

 The signal preprocessing steps are detailed in our previous Methods publication (Zondo et al. 2024). Due to journal space limitations, we could not expand on all signal pre-processing steps, and how we handled missing data for the current submission. We have however, revised the Methods section and included reference to the fNIRS pre-processing steps, and handling missing data.  

 With reference to reporting, we collected both oxygenated (HbO) and deoxygenated haemoglobin (HbR) signals but chose to focus on HbO for methodological and interpretative reasons. HbO has been shown to demonstrate a higher signal-to-noise ratio and greater sensitivity to task-evoked cortical activation in block-design paradigms, particularly in paediatric and adolescent samples. Similarly, we patterned our study on Khoe et al. 2020, who reported and analyzed HbO signal for easy replicability.****

**Optode Placement and Channel Analysis**- The description of fNIRS optode placement is vague. Based on the figures, it appears that 15 optodes (8 sources and 7 detectors) were positioned over:

- Prefrontal and premotor areas (e.g., S1- S4, D1–D4)
- Parietal regions (e.g., S5–S8, D6-D7)
- Interhemispheric regions (e.g., D4, D6) Please clarify which channels were analyzed and whether regions of interest (ROIs) were defined. Also, describe how data blocks were averaged for statistical analysis.

**Response:**

 Thank you. The montage consisted of 8 sources and 7 detectors arranged over the prefrontal cortex, (as detailed in Table 1 of the referenced protocol (Zondo et al.2024). No parietal regions were covered. Optode coverage extended across dorsolateral prefrontal, frontopolar, orbitofrontal, and adjacent premotor regions. Regions of interest (ROIs) were anatomically grouped into three predefined clusters: dorsolateral prefrontal cortex (DLPFC), frontopolar cortex (FPC), and orbitofrontal cortex (OFC), based on MNI coordinates and fOLD specificity mapping.

 All 22 channels were initially included in the analysis; however, channels were excluded if they failed scalp coupling index thresholds or signal quality criteria during preprocessing (using Satori). Data blocks were segmented using time-locked event markers and averaged per condition (congruent, incongruent) across each 10-second task block. Mean HbO amplitude within each block window was computed and aggregated at the ROI level (individual and group) for analysis.

**Results**

 The results section is generally well presented. However, the following improvements are suggested:

- Include significance values and effect sizes in Table 2 to enhance interpretability.

**Response**:

 We have since revised the table to include significance, test statistic, and effect sizes.

- Figures 2 and 3 do not clearly depict optode placement relative to the brain surface. Consider revising these to more accurately reflect spatial positioning.

**Response:**

 We did not use brain surface maps – all regions of interest configurations were determined using the fOLD software, and further tools inbuilt on Satori fNIRS. We do recognize this limitation, we are however confident, as determined by the fOLD data, (specificity indices) that optode placements were accurately confirmed.

- Table 3 raises questions about the anatomical labeling of optodes. For example:
  - Optodes S1–D3 and S6–D3 are used to represent the right dorsolateral prefrontal cortex (dlPFC), appearing anteriorly on the right side.
  - Optodes S8–D5 and S8–D7 are used for the left dlPFC but appear posteriorly on the left side. This discrepancy should be clarified.

**Response:**

 We did not use brain surface maps – all regions of interest configurations were determined using the fOLD software, and further inbuilt tools on Satori fNIRS. The configuration cap used in the study was further confirmed by NIRX technology for specificity.

- The manuscript does not mention any bad channels. Please confirm whether any channels were excluded due to poor signal quality.

**Response:**

 Thank you for the comment. As detailed in our MethodsX paper, the study was affected by bad channels, and poor scalp index coupling, primarily due to the population under study (skin pigmentation). We have however added this information to the methods section.  

**Discussion**

 The discussion is well articulated, but I encourage the authors to elaborate on the clinical implications of their findings. For instance, the emergence of significant between-group differences in hemodynamic responses during incongruent trials—despite limited behavioral effects is noteworthy and merits further exploration.

**Response:**

 We thank the reviewer for this comment. Within the Discussion section, we have sought to highlight the emergence of neural efficiency, concomitant with brain plasticity. Due to the small sample size, the clinical implications (at this stage), of significant hemodynamic differences on incongruent trials, is largely discussed in the context of emergent brain plasticity and neural efficiency. Our group is currently collecting Phase 2 data, to investigate the implications of the above findings at a clinical scale.  

 Additionally, the manuscript lacks a dedicated limitations section. Currently, limitations are briefly mentioned in the conclusion. I recommend restructuring the discussion to include a clear and comprehensive limitations section, followed by a conclusion that synthesizes key findings without introducing new concerns.

**Response:**

 We have since revised the manuscript and created a section detailing ‘Limitations’. Resulting from this revision, the ‘conclusion’, section is succinct, incorporating further suggestions from the other reviewers.
